# Ultrasonographic Diagnosis of Placenta Accreta Spectrum (PAS) Disorder: Ideation of an Ultrasonographic Score and Correlation with Surgical and Neonatal Outcomes

**DOI:** 10.3390/diagnostics11010023

**Published:** 2020-12-25

**Authors:** Valentina Del Negro, Natalia Aleksa, Cecilia Galli, Enrico Ciminello, Martina Derme, Flaminia Vena, Ludovico Muzii, Maria Grazia Piccioni

**Affiliations:** 1 Department of Maternal and Child Health and Urological Sciences, “Sapienza” University of Rome, 00161 Rome, Italy; valentina.delnegro@uniroma1.it (V.D.N.); aleksa.natalia@gmail.com (N.A.); cecilia.galli@uniroma1.it (C.G.); martina_derme@virgilio.it (M.D.); flaminia.vena@uniroma1.it (F.V.); ludovico.muzii@uniroma1.it (L.M.); 2 Department of Statistical Sciences, “Sapienza” University of Rome, 00185 Rome, Italy; enrico.ciminello@uniroma1.it

**Keywords:** caesarean section, placental pathology, diagnostic ultrasound, prenatal diagnosis

## Abstract

The objective of this study was to evaluate a novel ultrasonographic scoring system for the diagnosis of PAS and the prediction of maternal and neonatal outcomes. In this retrospective study, 138 patients with at least one previous caesarean section (CS) and placenta previa were included. They were divided into four groups ranging from Group 0 (Non PAS) to Group 3 (Placenta Percreta) according to the histological or surgical confirmation. Their ultrasound examinations during pregnancy were reviewed according to the nine different ultrasound signs reported by the European Working Group on Abnormally Invasive Placenta. For each parameter, 0 to 2 points were assigned. The sum of the points reflects the severity of PAS with a maximum score of 20. The scoring system revealed good performances in evaluation metrics, with an overall accuracy of 94%. In addition to this, patients’ characteristics and surgical and neonatal outcomes were analyzed with an evidence of higher incidence of complications in severe forms. Our study suggests that antenatal ultrasonographic diagnosis of PAS is feasible with sufficient level of accuracy. This will be important in identifying high-risk patients and implementing preventive strategy.

## 1. Introduction

Placenta accreta spectrum disorders, formerly known as pathologically adherent placenta, defines a group of conditions characterized by abnormal adhesion and/or invasion of the placental trophoblast to the uterine myometrium. The spectrum comprehends placenta accreta (adhesion of the placenta to myometrium without intervening decidua), placenta increta (infiltration of the trophoblast into the myometrium layer), and placenta percreta (infiltration through the myometrium, serosa, and eventually contiguous organs) [1].

It is widely recognized that there are two main risk factors for PAS: placenta previa and previous caesarean delivery. The significant rise of CS incidence has determined an important growth in the PAS prevalence, which has increased 13-fold since the early 1990s [2] and is now estimated to occur in approximately 3 in 1000 pregnancies [3].

PAS is a life-threatening condition. These patients, in fact, have an increased risk of severe hemorrhage frequently requiring blood transfusion. Moreover, both hysterectomy rates and maternal deaths are higher in this group of patients [4]. Severe hemorrhage, in fact, can lead to multisystem organ failure, disseminated intravascular coagulation (DIC), need for intensive care unit, hysterectomy, and even death [1]. In case of an appropriate antepartum diagnosis instead, the outcomes are definitely better and the patient can be adequately referred to a specialized center with a multidisciplinary team with adequate expertise [1].

To date, both ultrasound (US) and magnetic resonance imaging (MRI) have been used for the diagnosis of PAS [5]. However, according to FIGO consensus guidelines, US should be the first line technique for diagnosing PAS because of its low costs and wide availability. MRI is not strictly necessary for prenatal diagnosis of suspected PAS but can be an additional useful tool to determine pelvic extension of a placenta percreta or to better define doubtful regions on US [6].

A recent systematic review and meta-analysis by Jauniaux et al. has shown the high reliability of US in detecting PAS in women at high risk with a diagnostic accuracy of 90.9% [7]. Nevertheless, even in nations with widely applied prenatal diagnosis US screening, more than 50% of cases of PAS are not diagnosed before delivery [8].

In literature, many studies have proposed different diagnostic scores for PAS based upon ultrasonographic signs and/or clinical information with the purpose of predicting and diagnosing PAS and maternal-neonatal outcome [9,10,11,12,13,14,15,16,17,18]. However, the performances of these scores have shown considerable variability among all studies. In order to improve diagnostic accuracy, the ‘European Working Group on Abnormally Invasive Placenta’ has recently proposed a standardization of PAS imaging descriptors that have been unified under a common heading ultimately providing unambiguous definitions, with specific reference to the ultrasound modality [19].

The objective of our study is therefore to develop an ultrasound score in patients with risk factors for PAS using specific ultrasound markers defined by the European Working Group on Abnormally Invasive Placenta combined with anamnestic data (number of previous CS). The study aims to improve sensitivity, specificity and accuracy of 2D US in order to predict the severity of surgical and neonatal outcomes.

## 2. Materials and Methods

This retrospective single center study included pregnant women with at least one previous CS in anamnesis, who received an ultrasonographic diagnosis of placenta previa or low-lying placenta in the third trimester at our institute between January 2014 and November 2019. 

The diagnosis of placenta previa was based on the presence of placental tissue covering the internal cervical os whereas low-lying placenta was diagnosed when the placenta was within 2 cm from the internal cervical os but did not cover it. 

All patients enrolled signed a written informed consent. Patients with incomplete clinical and instrumental data and those who gave birth in another hospital were excluded. MRI was not performed in our study.

The primary endpoint of our study was to determine whether the different degrees of placental invasion (accreta, increta, percreta) were predictable and distinguishable from each other through the use of an ultrasonographic score. Secondary, all data regarding parity; age; obstetrical anamnesis; and the details of the preoperative, intraoperative, and postoperative treatments were collected in order to verify the validity of this score in predicting possible surgical complications and thus establishing the best therapeutic strategy.

The study was conducted according to the guidelines of the Declaration of Helsinki. Ethical review and approval were waived for this study, due to its retrospective design. Informed consent was obtained from all subjects involved in the study. Data is contained within the article or additional material file.

### 2.1. Conception of the Ultrasound Score

Nine different US signs previously reported by the European Working Group on Abnormally Invasive Placenta [19] and one anamnestic data (number of previous CS) were used to create our ultrasonographic score. Each parameter was stratified into three levels of severity identified respectively with 0, 1 or a maximum of 2 points. The sum of the points obtained from each parameter reflects the severity of PAS with a maximum score of 20. It is important to mention that each parameter included in our scoring system has previously been reported in literature to be strongly associated with morbidly adherent placenta. All parameters and their grades of severity are presented in Table 1.

1. Placental lacunae were graded with reference to Finberg’s study [20] for their number, the site (near or far from the myometrium), the shape (irregular, linear or round), the borders (echogenic or non-echogenic i.e., distinct or indistinct), and Doppler study (diffuse or focal turbulent or non-turbulent flow) as follows: score 0: absent; score 1: present in a number of 2–3, generally round and small, mean diameter 2 cm; score 2: present in number of 4–6, generally irregular, mean diameter 4 cm with turbulent blood flow or tributary vessels;
2. Retro placental space (Clear zone). Score 0: present; score 1: irregular; score 2: absent.
3. Retro placental myometrial thickness (millimeter). Score 0: myometrium >1 mm; score 1: myometrium <1 mm; score 2: myometrium not measurable (disappeared).
4. The bladder wall (hyperechoic uterine serosa-to-bladder interface). Score 0: line clear and complete; score 1: line vague or irregular; score 2: line lost.
5. Focal exophytic mass and/or placental bulge. Score 0 and score 1: absent; score 2: present.
6. Increased peri-uterine vascularity between uterus and urinary bladder. Score 0: normal flow; score1: increased flow, presence of numerous vases, tortuous; score 2: multidirectional flow or presence of bridge vessels.
7. Subplacental hypervascularity. Score 0: normal flow; score 1: increased flow; score 2: bridge vessels with perpendicular course
8. Diffuse or focal turbulent flow in the lacunae. Score 0: absent; score 1: focal; score 2: diffused with tributary vessels (>15 cm/sec)
9. Position of the placenta. Score 1: low lying; score 2: previa
10. The previous history of CS. Score 0: 1 previous CS; score 1: 2 previous CS; score 2: ≥3 previous CS.

### 2.2. Ultrasonographic Assessment

All patients enrolled were evaluated by a single expert operator (M.G.P.) using an ultrasound system equipped with a 4–8 MHz transabdominal transducer and a 5–9 MHz transvaginal transducer (Voluson 730, GE Medical Systems, Zipf, Austria). All images were collected in an electronic database. Subsequently, a highly-expert investigator, who was blinded to the final histological or surgical grading, reviewed all images evaluating each parameter of the score for each patient assigning 0 to 2 points on the basis of the classification created.

### 2.3. Clincal and Histopathologic Definition of Placental Invasion

The diagnosis of placental invasion was based on histological confirmation made by two expert pathologists. In all cases in which hysterectomy was avoided, the identification of adhesions was made during surgery by senior obstetricians from our institute according to FIGO classification for the clinical diagnosis of PAS disorders [21].

On the basis of histological examination and/or surgical grading determined according to FIGO guidelines [6], the patients were divided into four groups: Group 0 (Absence of PAS), Group 1 (Placenta Accreta), Group 2 (Placenta Increta), Group 3 (Placenta Percreta).

## 3. Results

During the 5 years of investigation, 138 patients met the inclusion criteria and were enrolled into our study. We firstly analyzed patients’ characteristics and risk factors for PAS according to each group by Kruskal–Wallis Test. Results are listed in Table 2. The mean maternal age did not differ in a statistically significant way between groups (*p* value < 0.357) such as the number of previous abortions (curettage) (*p* value < 0.587). On the contrary, the number of previous CS differed significantly between patients with PAS from those non-PAS (*p* value < 0.001). In group 3 (Placenta Percreta), all patients had placenta previa (*p* value < 0.001).

Ten parameters (listed in Table 1) were considered in order to build the ultrasonographic score used as a predictor of PAS. All those factors showed a strong dependence with the PAS group variable, according to Chi-Squared Test (*p* value < 0.001). Results are listed in Table 3.

We also analyzed the data related to placental location, anterior or posterior, to verify whether its distribution was related to the risk of placental accretion in our population. Data regarding placental localization are shown in Table 4. 

The hypothesis of independence between PAS and placental localization was rejected by Chi-squared test (*p* < 0.05), thus showing the possibility of a statistically significant relationship between PAS and anterior placental localization. 

### Surgical Outcomes

There was significant difference in the length of hospitalization among the groups: 9.5 days for group 3 versus 3 days for group 0. The duration of surgery in patients belonging to groups 2 and 3 was significantly longer (126.5–170 min versus 60 min in group 0) (*p* value < 0.001).

Relaparotomy occurred in two cases (10.5%) in group 1 due to uterine hemorrhage and in one case (25%) in group 3 due to pelvic abscess.

Access to the intensive care unit was greater in group 3 with three cases (75%) instead of one case (0.9%) in group 0 and one case (5.3%) in group 1 (*p* value < 0.001).

Blood transfusion requirements, the rate of emergency CS and recourse to interventional radiology were greater in patients’ groups with PAS versus patients without PAS, respectively (18 vs. 4, *p* value < 0.001), (27 vs. 15, *p* value < 0.01), (14 vs. 1, *p* < 0.001).

Subtotal hysterectomy was performed in four cases (21.1%) in group 1 and in two cases (33.3%) in group 2, whereas total hysterectomy was performed in 10 cases (52.6%) in group 1, in four cases (66.7%) in group 2 and in all cases (100%) in group 3. The difference among groups resulted to be statistically significant (*p* < 0.001).

The occurrence of DIC, the occurrence of ureteral and bladder lesions, and the use of Bakri Balloon did not differ statistically among groups. 

Surgical and Neonatal Outcomes are listed in Table 5.

The analysis for the creation of the score and the evaluation of its performances were conducted using the software R v. 3.6.3 (29 February 2020) “Holding the Windsock”.

**Figure 1:**
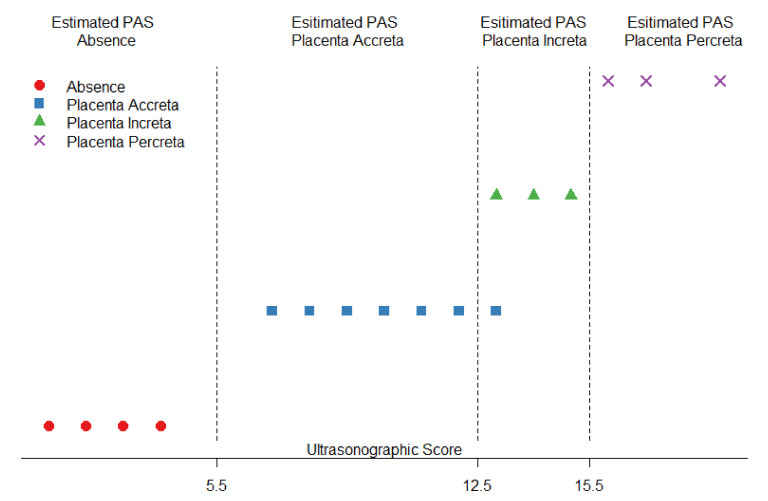
Estimated vs. actual diagnosis according to the proposed score.

The proposed score has a range of 0–20 and is created by the addition of the values of the 10 parameters presented in Table 1 for each patient. The domain 0–20 of the score is divided into four consecutive segments and, depending on where the value of the score falls, it corresponds to an estimate of a value of the PAS. The thresholds defining the segments are determined in order to optimize the classification power of the score, based on sensitivity and specificity for each class, resulting in the following structure: 0 < score < 5.5 the patient is estimated to belong to PAS group 0; 5.5 < score < 12.5 the patient is estimated to belong to PAS group 1; 12.5 < score < 15.5 implies the patient is estimated to belong to PAS group 2; 15.5 < score < 20 implies the patient is estimated to belong to PAS group 3 (Figure 1).

**Figure 2:**
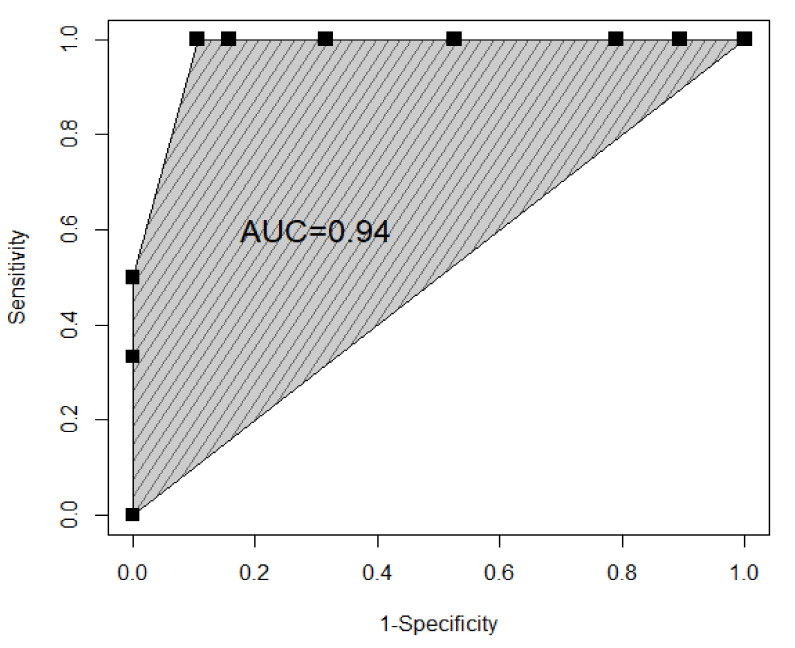
ROC curve for the classification of classes PAS group 1 and PAS group 2.

The score allows for perfect classification for external classes. In particular, when the score is below the threshold of 5.5, all patients actually belong to group 0 (Non PAS) whereas when the score is above 15.5, in all cases Placenta Percreta is actually diagnosed. On the other hand, as classes in the middle show an overlap of the estimates when the score is equal to 13 (Figure 1), it is worth reporting the evaluation metrics of the classification problem for these two classes: The analysis resulted in good performances for all the considered metrics, namely sensitivity of 100%, specificity of 89%, and accuracy of 92%, and provided the ROC curve in Figure 2, with an Area Under the Curve (AUC) score equal to 0.94.

## 4. Discussion

PAS is a pathologic anomaly of placental implantation in which the villous tissue adheres or invades the uterine wall leading to incomplete separation (partial or total) of the placental disc from the uterine wall at the time of delivery. The main effect is the possibility of a massive hemorrhage, especially when the pathology is not known at the time of delivery, with, consequently, important effects on maternal and fetal mortality and morbidity [22]. In our sample population, post-partum complications (i.e., the use of Bakri-Balloon and uterine artery embolization) as well as surgery duration and rates of hysterectomy increased with the severity of PAS. Patients with placenta percreta had a larger amount of intraoperative blood loss, major request for blood transfusion and higher incidence of bladder and ureteral lesions.

Previous caesarean delivery and placenta previa are the two main risk factors for PAS and because of the significant rise of caesarean delivery rate, PAS is becoming more and more frequent [3]. Even though the definitive diagnosis of PAS is generally made on the expelled placenta, prenatal US diagnosis is becoming increasingly common and clinically relevant [23], allowing accurate planning of delivery, so decreasing the incidence of adverse events. Nevertheless, PAS is still not recognized prenatally in a percentage of cases ranging from 50–70% [22]. Many studies published so far have proposed different ultrasonographic signs evocative of PAS [9,10,11,12,13,14,15,16,17,18]. Anyway, these studies frequently use the same descriptors with different names or, on the contrary, the same definition has been used for different finds. Moreover, each study reports great variability in US signs sensibility and specificity. 

Finally, in 2016, the ‘European Working Group on Abnormally Invasive Placenta’ proposed a standardization of PAS descriptors based on a systematic review of 23 previous studies [19]. In our study, we found that a score derived from the ultrasound descriptors standardized by the ‘European Working Group on Abnormally Invasive Placenta’, each one stratified into three levels of severity, combined with one anamnestic data (number of previous CS), was sufficiently predictive of placental invasion among pregnancies at increased risk.

Our scoring system for predicting PAS showed sensitivity of 100%, specificity of 89% and accuracy of 92%. The above-mentioned data demonstrates that our scoring system for predicting PAS and the subsequent degree of risk is sufficiently reliable. According to different scoring levels, a customized follow-up and treatment options for patients can be planned in order to reduce the potential maternal and children morbidity and mortality.

In the future, we will have the possibility to organize a multidisciplinary and experienced team, including obstetrics, pediatricians, urologists, vascular surgeons, anesthesiologists, and laboratory doctors to perform corresponding treatment measures and blood preparation, reducing the potential risk of maternal morbidity and mortality.

The main limitations of our study were the retrospective single institution study design, the small cohort size and the poor applicability of our model to low-risk pregnancies, because it was developed only for high-risk pregnancies in the third trimester (patients with at least one previous CS and placenta previa). Strengths of our paper are represented by: blinded review process, the use of histologic confirmation and the use of standardized US descriptors, all with a validity already confirmed by the literature. 

## 5. Conclusions

Patients with PAS have higher risk of severe complications. It is strongly necessary to ameliorate the diagnostic accuracy of PAS in order to improve both maternal and neonatal outcomes. The predictive accuracy of this model was sufficiently high and this lays the foundation for the prediction of PAS. Anyway, there are some limitations to this study: The main are that this was a retrospective single-center study and the sample size was relatively small. Further multicenter prospective studies are needed to test the scoring system on larger population and to definitely validate its applicability in clinical practice.

## Figures and Tables

**Table 1:** Ultrasonographic Score.

Ultrasound and Clinical Signs	Score = 0	Score = 1	Score = 2
Placental lacunae	Not seen	2–3, regular ≤2 cm	4–6, irregular, 4 cm
Hypoechoic retroplacental space ("clear zone")	Present	Irregular	Absent
Myometrial thinning	Myometrium > 1 mm	Myometrium < 1 mm	Absent
Hyperechoic uterus–bladder interface (bladder line)	Line clear and complete	Line vague or irregular	Line lost
Focal exophytic mass and/or placental bulge	Absent	-	Present
Utero-vescical hypervascularity	Absent	Increased	Multidirectional flow with bridging vessels
Prior Caesarean section	1	2	≥3
Placental relationship with internal cervical os	-	Low-lying	Previa
Subplacental hypervascularity	Normal	Increased with numerous vases, tortuous	Bridging vessels with perpendicular course
Diffuse or focal turbulent flow in the lacunae	Absent	Focal turbulent flow	Diffuse turbulent flow with feeding vessels

**Table 2:** Patients’ characteristics and risk factors for PAS.

Characterics and Risk Factors	Normal Placenta	Placenta Accreta	Placenta Increta	Placenta Percreta	*p* Value
	Group 0	Group 1	Group 2	Group 3	
Number of patients	109	19	6	4	
Age (years) mean age ± SD	38 (±5.4)	39 (±5.18)	40 (±6.9)	35 (±5.25)	<0.357 ^a^
Previous CS 0 (1)	99 (90.8%)	3 (15.8%)	1 (16.7%)	0 (0%)	<0.001 ^a^
Previous CS 1 (2)	6 (5.5%)	12 (63.2%)	3 (50%)	1 (25%)	<0.001 ^a^
Previous CS 2 (≥3)	4 (3.7%)	4 (21.1%)	2 (33.3%)	3 (75%)	<0.001 ^a^
Placenta Previa	40 (36.7%)	11 (58%)	4 (66.7%)	4 (100%)	<0.001 ^b^
Low-lying Placenta	69 (63.3%)	8 (42%)	2 (33.3%)	0 (0%)	<0.001 ^b^
Previous curettage	26 (72.2%)	6 (16.7%)	2 (5.6%)	2 (5.6%)	< 0.587 ^b^

*p* < 0.001 was considered statistically significant; ^a^ Values given as median (interquartile range), Kruskal–Wallis test; ^b^ Values given as *n* (%), Chi-squared test; CS, caesarean section; Significant differences between: without PAS and PAS.

**Table 3:** Parameters predictive of PAS.

Ultrasound and Clinical Markers	Group 0 Without PAS *n* 109 (79%)	Group 1 PA *n* 19 (13.7%)	Group 2 PI *n* 6 (4,3%)	Group 3 PP *n* 4(3,0%)	*p* Value
Placental lacunae					
0	109 (100%)	0	0	0	0.001
1		19 (100%)	0	0	0.001
2	0	0	6 (100%)	4 (100%)	0.001
Hypoechoic retroplacental space ("clear zone")					
0	109 (100%)	0	0	0	0.001
1	0	5 (26.3 %)	0	0	0.001
2	0	14 (73.7%)	6 (100%)	4 (100%)	0.001
Myometrial thickness					
0	106 (97.2%)	0	0	0	0.001
1	3 (2.8%)	12 (63.2%)	0	0	0.001
2	0	7 (36.8%)	6 (100%)	4 (100%)	0.001
Bladder line					
0	109% (100%)	19 (100%)	1 (17%)	0	0.001
1	0	0	5 (83%)	0	0.001
2	0	0	0	4 (100%)	0.001
Focal exophytic mass or placental bulging					
0	109 (100%)	19 (100%)	6 (100%)	1 (25%)	0.001
2	0	0	0	3 (75%)	0.001
Vascularity at the uterus–bladder interface					
0	109 (100%)	19(100%)	3 (50%)	0	0.001
1	0	0	3 (50%)	0	0.001
2	0	0	0	4 (100%)	0.001
Subplacental hypervascularity					
0	109 (100%)	0	0	0	0.001
1	0	11 (57.9%)	0	0	0.001
2	0	8 (42.1%)	6 (100%)	4 (100%)	0.001
Diffuse or focal turbulent flow in the lacunae					
0	109 (100%)	0	0	0	0.001
1	0	9 (47.4%)	2 (33.3%)	0	0.001
2	0	10 (52.6%)	4 (66.7%)	4 (100%)	0.001
The previous history of caesarean section					
0	99 (90.8%)	3 (15.8%)	1 (16.7%)	0 (0%)	0.001
1	6 (5.5%)	12 (63.2%)	3 (50%)	1 (25%)	0.001
2	4 (3.7%)	4 (21.1%)	2 (33.3%)	3 (75%)	0.001
Placental relationship with internal cervical os					
1	69 (63.3%)	8 (42.0%)	2 (33.3%)	0 (0%)	0.001
2	40 (36.7%)	11 (58.0%)	4 (66.7%)	4 (100%)	0.001

PA—placenta accreta, PI—placenta increta, PP—placenta percreta; Values given as frequency (percentage), Chi-squared test.

**Table 4:** Placental localization.

Placental Localization	Group 0 Without PAS *n* 109	Group 1 PA *n* 19	Group 2 PI *n* 6	Group 3 PP *n* 4
Anterior placenta	104	15	6	3
Posterior placenta	5	4	0	1

Pearson Chi-Squared Test. Chi-Squared test statistics = 8.886. *p* value = 0.03.

**Table 5:** Surgical and neonatal outcomes according to the score of PAS.

Outcomes	Without PAS		PAS						*p* Value
	Group 0, without PAS, Score ≤5	*n*	Group 1, P. Accreta, Score >5–<12.5	*n*	Group 2, P. Increta, Score >12.5–≤15.5	*n*	Group 3, P. Percreta, Score >15.5 –≤20	*n*	
Length of hospital stay (day) ± SD	4 ± 2.3	109	6 ± 4.5	19	8.5 ± 5.6	6	9.5 ± 3.9	4	<0.001
Duration of surgery (min) ± SD	60 ± 16.6	109	100 ± 36.3	19	126.5 ± 21.4	6	170 ± 84.2	4	<0.001
Admission to intensive care unit	0.90%	1	5.30%	1	0%	0	75%	3	<0.001
Transfusion ≥ 4 PRBC	3.60%	4	68.40%	13	33.30%	2	75%	3	<0.001
Transfusion of Plasma	1.83%	2	47.30%	9	33.30%	2	75%	3	<0.001
Interventional radiology request	-	-	-	-	-	-	-	-	-
Re-laparotomy	0%	0	10.50%	2	0%	0	0%	0	<0.001
Hysterectomy:		-	-	-	-	-	-	-	<0.001
Subtotal	0%	0	21.00%	4	33.30%	2	-	-	
Total	0.90%	1	52.60%	10	66.70%	4	100%	4	
DIC	0.90%	1	10.50%	2	0%	0	75%	3	<0.064
Bladder lesion	0%	0	5.30%	1	0%	0	75%	3	<0.001
Ureteral lesion	0%	0	0%	0	0%	0	50%	2	<0.001
Urgency Caesarean section	24.80%	27	52.60%	10	33.30%	2	75%	3	<0.001
Balloon tamponade Bakri	12.80%	14	36.80%	7	0%	0	0%	0	<0.008
General anesthesia	0.90%	1	10.50%	2	0%	0	50%	2	<0.009
Apgar score 5 min (minim/max)	9 (7–10)	109	9 (7–10)	19	8 (7–10)	6	7 (7–8)	4	<0.013
Gestational age at delivery ± SD	36 ± 2.5	109	34 ± 2.9	19	33 ± 1.89	6	32.5 ± 2.63	4	<0.001
Birth weight (gr)	2680	109	2160	19	1980	6	1870	4	<0.001

Values given as frequency (*n*) (%), Chi-squared test b; PRBC number of units; Building and assessment of the Score.
